# Relations in the context of Turiyam sets

**DOI:** 10.1186/s13104-023-06292-4

**Published:** 2023-04-09

**Authors:** Gamachu Adugna Ganati, V. N. Srinivasa Rao Repalle, Mamo Abebe Ashebo

**Affiliations:** Department of Mathematics, Wollega University, Nekemte, Ethiopia

**Keywords:** Turiyam sets, Neutrosophic sets, Turiyam relations, 03B52, 03E20

## Abstract

**Objective:**

Recently, the Turiyam set was introduced as an extension of the neutrosophic set to handle the uncertainty data set beyond its truth, indeterminacy and falsity values. This article introduced the Cartesian product of Turiyam sets and Turiyam relations. Further, we defined operations on Turiyam relations as well as discussed the inverse and types of Turiyam relations.

**Results:**

The Cartesian product of Turiyam sets, Turiyam relations, inverse Turiyam relation and types of Turiyam relations are stated and their properties are derived. Furthermore, examples are given to clarify some concepts.

## Introduction

In 1998 neutrosophic set (NS) theory was developed by Smarandache as a mathematical tool to handle a situation involving indeterminacy, imprecise, and inconsistent information [1]. The researcher introduced this concept by adjusting the concepts of fuzzy set [2] and intuitionistic fuzzy set [3]. Each element in the NS is determined by membership value, unknown value, and non-membership value and those three values are independent of each other [1]. Due to its flexibility and effectiveness, this set is applied in different situations by many researchers worldwide [4]. For example, in [4–9], researchers studied applications of NS in decision making, medical diagnosis, image processing, economics, computer science, and so on. In 2013, the refined neutrosophic set was also developed by Smarandache to handle n-valued information by using the neutrosophic components [10]. But the problem arises when uncertainty exists beyond truth, indeterminacy and falsity values [11]. For instance, in the case of COVID 19 we have four dimensions, i.e., recovered, active, death, and vaccinated cases [11]. Due to the fourth dimension, the neutrosophic set didn’t represent the COVID 19 data precisely [11]. The plithogenic set was developed by Smarandache as the generalization of NS and try to handle this situation by considering it as a contradiction rather than taking it as a new dimension [12]. However, to deal with such situations, a well-known Indian researcher motivated by ontological theory found in Sanskrit developed a new mathematical concept called the Turiyam set (TS) [11, 13, 14]. This set is the extension of NS in which its elements are determined by membership values (*t*), unknown values (*i*), non-membership values (*f*), and liberal values (*l*) in a universal set $U$ and is referred to as $T = \left\{ { \left\langle {x:t,i,f,l} \right\rangle :x \in U} \right\}$ [14]. Those four dimensions of this set are independent and lie in [0, 1] such that $0 \le t + i + f + l \le 4$ [11]. This set is applicable in areas like sports data, chemistry, the arts, medical diagnosis, voting systems, and so on [11, 13, 14]. The existing literature shows there is no relation defined in the context of Turiyam sets. Thus, this research paper introduces the relations on Turiyam sets as the generalization of neutrosophic relations [15, 16] and then derives some of their properties. In this regard, four dimensional logic [17] and its algebra [18] are required.

The next part of this paper is arranged as follows: first, we collect some preliminary concepts. Second, we apply the concept of relations to Turiyam sets. Third, we describe the types of Turiyam relations. Finally, we give the conclusion followed by future work recommendation.

## Main text

First, we collect concepts that are useful for this work [1, 13, 14].

Consider U is a universe set.

### **Definition 1**

[1] A NS $A$ on $U \ne \emptyset$ has the form $A = \left\{ {\left\langle {x, T_{A} \left( x \right), I_{A} \left( x \right), F_{A} \left( x \right)} \right\rangle :x \in U} \right\}$,where ${\text{T}}_{A} \left( x \right):U \to \left] {^-0,1^{ + } } \right[, I_{A} \left( x \right):U \to \left] {^-0,1^{ + } } \right[$. and $F_{A} \left( x \right):U \to \left] {^-0,1^{ + } } \right[$ denote the truth value, the indeterminacy value and the falsity value for each $x \in X$ correspondingly by which ${\text{T}}_{A} \left( x \right), I_{A} \left( x \right)$ and $F_{A} \left( x \right)$ satisfies the condition $^-0\le {\text{T}}_{A} \left( x \right) + I_{A} \left( x \right) + F_{A} \left( x \right) \le 3^{ + } ,\forall x \in U.$

### **Definition 2**

[13, 14] A Turiyam set $B$ on $U \ne \emptyset$ has the form

$$B = \left\{ {\left\langle {x, t_{B} \left( x \right), i_{B} \left( x \right), f_{B} \left( x \right),l_{B} \left( x \right)} \right\rangle :x \in U} \right\}.$$

where ${\text{t}}_{B} \left( x \right):U \to \left[ {0,1} \right], i_{B} \left( x \right):U \to \left[ {0,1} \right]$, and $l_{B} \left( x \right):U \to \left[ {0,1} \right]$ denote the truth value, the indeterminacy value, the falsity value and the Turiyam state (or liberal) value for each $x \in X$, correspondingly by which ${\text{t}}_{B} \left( x \right), i_{B} \left( x \right)$, $f_{A} \left( x \right)$ and $l\left( x \right)$ satisfies the condition $^-0 \le t_{B} \left( x \right) + i_{B} \left( x \right) + f_{B} \left( x \right) + l_{B} \left( x \right) \le 4^{ + } ,\forall x \in U.$Furthermore, the empty Turiyam set andhe universal Turiyam set are defined as $\emptyset_{T} = \left( {t,i,f,0} \right)$ and $U_{T} = \left( {t,i,f,1} \right)$ respectively.

### *Remark*

$1 - \left( {t + i + f + l} \right)$ is called the refusal degree of Turiyam sets.

We write Turiyam sets on universe set on U by TS (U).

### **Definition 3**

[14] Let A and B be two Turiyam sets on universe set U.

- A is said to be Turiyam subset of B if ${\text{t}}_{A} \left( x \right) \le {\text{t}}_{B} \left( x \right), i_{B} \left( x \right) \le i_{B} \left( x \right), f_{A} \left( x \right) \ge f_{B} \left( x \right)$, and $l_{A} \left( x \right) \le l_{B} \left( x \right),\forall x \in U.$
- A and B are equal if $A \subseteq B\;{\text{and}}\;B \subseteq A$.

### **Definition 4**

[14] Let A and B be two Turiyam sets on universe set U.

- The complement of A (denoted by $A^{c}$) is defined as: for all $x \in U, t^{c} \left( x \right) = f\left( x \right),i^{c} \left( x \right) = 1 - i\left( x \right),f^{c} \left( x \right) = t\left( x \right), l^{c} \left( x \right) = 1 - \left( {t\left( x \right) + i\left( x \right) + f\left( x \right)} \right)$
- The union of A and B (denoted by ${\text{A}} \cup B$) defined as $A \cup B = \left\{ {t_{A} \vee t_{B} ,i_{A} \wedge i_{B} ,f_{A} \wedge f_{B} ,l_{A} \vee l_{B} } \right\}$ where $(t_{A} \vee t_{B} )\left( x \right) = t_{A} \left( x \right) \vee t_{B} \left( x \right), (i_{A} \wedge i_{B} )\left( x \right) = i_{A} \left( x \right) \wedge i_{B} \left( x \right),(l_{A} \vee l_{B} )\left( x \right) = l_{A} \left( x \right) \vee l_{B} \left( x \right), \forall x \in U.$
- The intersection A and B (denoted by ${\text{A}} \cap B$) defined as ${\text{A}} \cap B = \left\{ {t_{A} \wedge t_{B} ,i_{A} \vee i_{B} ,f_{A} \vee f,l_{A} \wedge l_{B} } \right\}$.

Notice that the operators $\vee$ and $\wedge$ represent maximum and minimum respectively.

## Turiyam relations

In this section, first we define the Cartesian products of Turiyam sets. Based on this concept, we introduce relation on Turiyam sets and we give some types of it.

Let A, B, C $$\ne \emptyset \in TS\left( U \right).$$

### **Definition 5**

Let A, B $\ne \emptyset \in TS\left( U \right).$ The Cartesian product of A and B (denoted by $A \times B$) is a Turiyam set in $U \times U$ given by

$$A \times B = \left\{ {\left\langle {\left( {x,y} \right), t_{A \times B} \left( {x,y} \right),i_{A \times B} \left( {x,y} \right),f_{A \times B} \left( {x,y} \right),l_{A \times B} \left( {x,y} \right) > :\left( {x,y} \right) \in A \times B} \right\rangle } \right\}$$

where $t_{A \times B} ,i_{A \times B} ,f_{A \times B} ,l_{A \times B} :U \to \left[ {0,1} \right]$ such that

$$\begin{aligned} t_{A \times B} \left( {x,y} \right) & = \min \left\{ {t_{A} \left( x \right),t_{B} \left( y \right)} \right\} \\ i_{A \times B} \left( {x,y} \right) & = \min \left\{ {i_{A} \left( x \right),i_{B} \left( y \right)} \right\} \\ f_{A \times B} \left( {x,y} \right) & = \max \left\{ {f_{A} \left( x \right),f_{B} \left( y \right)} \right\} \\ l_{A \times B} \left( {x,y} \right) & = \min \left\{ {l_{A} \left( x \right), l_{B} \left( y \right)} \right\}. \\ \end{aligned}$$

### *Example 1*

Let $U = \left\{ {a, b,c} \right\}$ be a universe set. Let $A = \left\{ {\left\langle {a, \left( {0.1, 0.3, 0.2, 0.4} \right)} \right\rangle ,\;\left\langle {b, \left( {0.0, 0.2, 0.1, 0.3} \right)} \right\rangle } \right\}$ and

$$B = \left\{ {\left\langle {a, \left( {0.0, 0.2, 0.1, 0.5} \right)} \right\rangle ,\left\langle {b, \left( {0.3, 0.4, 0.1,1} \right)} \right\rangle ,\left\langle {c,\left( {0.2,0.4,0.3,0.1} \right)} \right\rangle } \right\}$$

be Turiyam sets on U. Then, $A \times B = \left\{ {\left\langle {\left( {a,a} \right), \left( {0.0, 0.2, 0.2,0.4} \right)} \right\rangle ,} \right.$

$$\left. {\left\langle {\left( {a,b} \right), \left( {0.1, 0.3, 0.2, 0.4} \right)} \right\rangle ,\left\langle {\left( {a,c} \right), \left( {0.1,0.3, 0.3,0.1} \right)} \right\rangle < \left( {b,a} \right), (0.0, 0.2, 0.1, 0.3 > , < \left( {b,b} \right), (0.0, 0.2, 0.1, 0.3 > ,\left\langle {\left( {b,c} \right),\left( {0.0,0.2,0.3,0.1} \right)} \right\rangle } \right\}$$

### **Definition 6**

Let $\emptyset \ne$ A, B $\in TS\left( U \right)$. Then a relation from A to B is a Turiyam subset of $A \times B$ which has the form ${\text{R}} = \left\{ {t_{R} , i_{R} ,f_{R} , l_{R} } \right\}$ where $t_{R} , i_{R} ,f_{R} , l_{R} : A \times B \to \left[ {0,1} \right]$ denote the truth membership function, indeterminacy membership, falsity membership function and liberation membership function respectively.

### *Example 2*

Consider Example 1 above

- $$R_{1} = \left\{ {\left\langle {(a,a), \left( {0.0, 0.2, 0.4, 0.3} \right)} \right\rangle , \left\langle {\left( {a,b} \right), \left( {0.1, 0.2, 0.3, 0.0} \right)} \right\rangle,} \right.$$$$\left. {\left\langle {\left( {b,a} \right),\left( {0.0, 0.1, 0.3, 0.2} \right)} \right\rangle ,\left\langle {\left( {b,b} \right), \left( {0.0, 0.0, 0.2, 0.3} \right)} \right\rangle } \right\}$$ and
  $$R_{2} = \left\{ {\left\langle {(a,a), \left( {0.0, 0.1, 0.3, 0.4} \right)} \right\rangle ,\left\langle {\left( {a,b} \right), \left( {0.0, 0.1, 0.3, 0.2} \right)} \right\rangle,} \right.$$$$\left. { \left\langle {\left( {b,a} \right),\left( {0.0, 0.2, 0.3, 0.1} \right)} \right\rangle ,\left\langle {\left( {b,b} \right), \left( {0.0, 0.1, 0.3, 0.1} \right)} \right\rangle ,\left\langle {\left( {b,c} \right),\left( {0.0,0.2,0.4,0.0} \right)} \right\rangle } \right\}$$are Turiyam relations from A to B on U.
- $$R_{3} = \left\{ {\left\langle {(a,a), \left( {0.0, 0.2, 0.6, 0.3} \right)} \right\rangle , \left\langle {\left( {a,b} \right), \left( {0.2, 0.3, 0.8, 0.7} \right)} \right\rangle,} \right.$$$$\left. { \left\langle {\left( {b,a} \right),\left( {0.0, 0.1, 0.5, 0.2} \right)} \right\rangle ,\left\langle {\left( {b,b} \right), \left( {0.3, 0.0, 0.5, 0.3} \right)} \right\rangle } \right\}$$ is not a relation from A to B.

### **Definition 7**

Let R: ${\text{A}} \to B$ be a Turiyam relation on U. The domain and range of R is defined as

$$Dom \left( R \right) =$$$$\left\{ {\left\langle {a,t\left( a \right),i\left( a \right),f\left( a \right),l\left( a \right)} \right\rangle \in {A \mathord{\left/ {\vphantom {A {\left\langle {\left( {a,b} \right), t\left( {a,b} \right),i\left( {a,b} \right),f\left( {a,b} \right),l\left( {a,b} \right)} \right\rangle }}} \right. \kern-0pt} {\left\langle {\left( {a,b} \right), t\left( {a,b} \right),i\left( {a,b} \right),f\left( {a,b} \right),l\left( {a,b} \right)} \right\rangle }} \in R} \right\}$$ and $$Ran\left( R \right) =$$$$\left\{ {\left\langle {b,t\left( b \right),i\left( b \right),f\left( b \right),l\left( b \right)} \right\rangle \in {B \mathord{\left/ {\vphantom {B {\left\langle {\left( {a,b} \right),t\left( {a,b} \right),i\left( {a,b} \right),f\left( {a,b} \right),l\left( {a,b} \right)} \right\rangle }}} \right. \kern-0pt} {\left\langle {\left( {a,b} \right),t\left( {a,b} \right),i\left( {a,b} \right),f\left( {a,b} \right),l\left( {a,b} \right)} \right\rangle }} \in R} \right\}$$ respectively.

### *Example 3*

Consider Example 2. Then

$$\begin{aligned} {\text{Dom }}(R_{1} ) & = \left\{ {\begin{array}{*{20}c} {\left\langle {a,\left( {0.1, 0.3, 0.2, 0.4} \right)} \right\rangle ,} \\ {\left\langle {b,\left( {0.0, 0.2, 0.1, 0.3} \right)} \right\rangle } \\ \end{array} } \right\} \\ {\text{and}}\;{\text{Ran}}(R_{2} ) & = \left\{ {\left\langle {a,\left( {0.0, 0.2, 0.1, 0.5} \right)} \right\rangle ,\left\langle {{\text{b}},\left( {0.3, 0.4, 0.1,1} \right)} \right\rangle ,\left\langle {{\text{c}},\left( {0.2,0.4,0.3,0.1} \right)} \right\rangle } \right\}. \end{aligned}$$

### **Definition 8**

Let R:${\text{A}} \to B$ be a Turiyam relation on U. Then,

- The Turiyam relation $R^{ - 1} :B \to A$ is the inverse of R and defined as $R^{ - 1} \left( {x,y} \right) = R\left( {y,x} \right),\;{\text{that is}},\; T_{{R^{ - 1} }} \left( {x,y} \right) = T_{R} \left( {y,x} \right), I_{{R^{ - 1} }} \left( {x,y} \right) = I_{R} \left( {y,x} \right)$ and $L_{{R^{ - 1} }} \left( {x,y} \right) = L_{R} \left( {y,x} \right),\forall \left( {x,y} \right) \in R$
- The complement of R (denoted by $R^{C}$) is defined as
  $$R^{C} = \left\{ {T_{{R^{C} }} , I_{{R^{C} }} ,F_{{R^{C} }} , L_{{R^{C} }} } \right\}$$ where $$T_{{R^{C} }} \left( {x,y} \right) = F_{R} \left( {x,y} \right),I_{{R^{C} }} \left( {x,y} \right) = 1 - I_{R} \left( {x,y} \right), F_{{R^{C} }} \left( {x,y} \right) = T_{R} \left( {x,y} \right)$$ and $$L_{{R^{C} }} \left( {x,y} \right) = 1 - \left( {T_{R} \left( {x,y} \right) + I_{R} \left( {x,y} \right) + F_{R} \left( {x,y} \right)} \right)$$.

### *Example 4*

Consider Example 2. Then

- $$R_{1}^{c} = \left\{ {\left\langle {(a,a), \left( {0.4, 0.8, 0.0, 0.4} \right)} \right\rangle ,\left\langle {\left( {a,b} \right), \left( {0.3, 0.8, 0.1,0.4} \right)} \right\rangle ,\left\langle {\left( {b,a} \right),(\left( {0.3, 0.9, 0.0, 0.6} \right)} \right\rangle ,\left\langle {\left( {b,b} \right), \left( {0.2, 1, 0.0, 0.8} \right)} \right\rangle } \right\}$$
- $${\text{R}}_{2}^{ - 1} = \left\{ {\left\langle { < ({\text{a}},{\text{a)}},\left( {0.0, 0.1, 0.3, 0.4} \right)} \right\rangle ,\left\langle {\left( {{\text{a}},{\text{b}}} \right),\left( {0.0, 0.2, 0.3, 0.1} \right)} \right\rangle ,\left\langle {\left( {{\text{b}},{\text{a}}} \right),\left( {0.0,0.1,0.9,0.5} \right)} \right\rangle ,\left\langle {\left( {{\text{b}},{\text{b}}} \right),\left( {0.0, 0.1, 0.3, 0.1} \right)} \right\rangle ,\left\langle {\left( {{\text{b}},{\text{c}}} \right),\left( {0.0,0.2,0.4,0.1} \right)} \right\rangle } \right\}$$

### **Theorem 1**

*Let* $R_{1} \;{and}\;R_{2}$ *be two Turiyam relations. Then,*

- $$(R_{2}^{ - 1} )^{ - 1} = R_{2}$$ *and* $$\left( {R_{1}^{c} } \right)^{c} = R_{1}$$
- $$R_{1} \subseteq R_{2} \Rightarrow R_{1}^{ - 1} \subseteq R_{2}^{ - 1}$$
- $$\left( {R_{1} \cup R_{2} } \right)^{ - 1} = R_{1}^{ - 1} \cup R_{2}^{ - 1}$$ *and* $$\left( {R_{1} \cap R_{2} } \right)^{ - 1} = R_{1}^{ - 1} \cap R_{2}^{ - 1}$$
- $$\left( {R_{1} \cup R_{2} } \right)^{c} = R_{1}^{c} \cap R_{2}^{c}$$ *and* $$\left( {R_{1} \cap R_{2} } \right)^{c} = R_{1}^{c} \cup R_{2}^{c}$$

### *Proof*

Easily, (a)–(d) hold.

### **Definition 9**

Let $R_{1}$ and $R_{2}$ be two Turiyam relations on universe set U.

- $R_{1}$ is said to be Turiyam subset of $R_{2}$ if ${\text{t}}_{{R_{1} }} \left( {x,y} \right) \le {\text{t}}_{{R_{2} }} \left( {x,y} \right), i_{{R_{1} }} \left( {x,y} \right) \le i_{{R_{2} }} \left( {x,y} \right), f_{{R_{1} }} \left( {x,y} \right) \ge f_{{R_{2} }} \left( {x,y} \right)$, and $l_{R_1} \left( {x,y} \right) \le l_{{R_{2} }} \left( {x,y} \right),\forall \left( {x,y} \right) \in U \times U.$
- $$R_{1} = R_{2}$$ if $$R_{1} \subseteq R_{2} \;{\text{and}}\;R_{2} \subseteq R_{1}$$
- The union of $$R_{1}$$ and $$R_{2}$$ is given by $$R_{1} \cup R_{2} =$$$$\left\{ {\left\langle {\left( {x,y} \right), {\text{t}}_{{R_{1} }} \left( {x,y} \right) \vee {\text{t}}_{{R_{2} }} \left( {x,y} \right),{\text{i}}_{{R_{1} }} \left( {x,y} \right) \wedge {\text{i}}_{{R_{2} }} \left( {x,y} \right),{\text{f}}_{{R_{1} }} \left( {x,y} \right) \wedge {\text{f}}_{{R_{2} }} \left( {x,y} \right),l_{{R_{1} }} \left( {x,y} \right) \vee l_{{R_{2} }} \left( {x,y} \right)} \right\rangle } \right.$$$$\left. {:\forall \left( {x,y} \right) \in U \times U} \right\}.$$
- The intersection of $R_{1}$ and $R_{2}$ is given as
  $$R_{1} \cap R_{2} = \left\{ {\left\langle {\left( {x,y} \right), {\text{t}}_{{R_{1} }} \left( {x,{\text{y}}} \right) \wedge {\text{t}}_{{R_{2} }} \left( {x,{\text{y}}} \right),{\text{i}}_{{R_{1} }} \left( {x,{\text{y}}} \right) \wedge {\text{i}}_{{R_{2} }} \left( {x,{\text{y}}} \right),{\text{f}}_{{R_{1} }} \left( {x,{\text{y}}} \right) \vee {\text{f}}_{{R_{2} }} \left( {x,{\text{y}}} \right), l\left( {x,{\text{y}}} \right) \wedge l_{{R_{2} }} \left( {x,{\text{y}}} \right)} \right\rangle :\forall \left( {x,y} \right) \in U \times U} \right\}$$
  Remark: Let R be a relation on universe set U. Then,
- R is called a null Turiyam relation if $l_{R} \left( {x,y} \right) = 0$ and $t_{R} \left( {x,y} \right) = i_{R} \left( {x,y} \right) = f_{R} \left( {x,y} \right) = 1,$
  $$\forall \left( {x,y} \right) \in U.$$
- R is called an absolute Turiyam relation if $l_{R} \left( {x,y} \right) = 1$ and $t_{R} \left( {x,y} \right) = i_{R} \left( {x,y} \right) = f_{R} \left( {x,y} \right) = 0, \forall \left( {x,y} \right) \in U.$

## Composition of Turiyam relations

### **Definition 10**

Let $R_{1} :A \to B$ and $R_{2} :B \to C$ be two Turiyam relations on $U$. The composition of $R_{1}$ and $R_{2}$ is a Turiyam relation $R_{2} \circ R_{1} :A \to C$ on $U$ defined as

$$\begin{aligned} & R_{2} \circ R_{1} = \left\{ {t_{{ R_{2} \circ R_{1} }} ,i_{{ R_{2} \circ R_{1} }} , f_{{ R_{2} \circ R_{1} }} ,l_{{ R_{2} \circ R_{1} }} } \right\} {\text{where}}\;\\ & t_{{ R_{2} \circ R_{1} }} \left( {a,c} \right) = \max_{b\in B}\left\{ {\min \left[ {t_{{ R_{1} }} \left( {a,b} \right),t_{{ R_{2} }} \left( {b,c} \right)} \right]} \right\}; \\ \end{aligned}$$

$$i_{{ R_{2} \circ R_{1} }} \left( {a,c} \right) = \min_{b\in B}\left\{ {\max \left[ {t_{{ R_{1} }} \left( {a,b} \right),t_{{ R_{2} }} \left( {b,c} \right)} \right]} \right\};$$

$$f_{{ R_{2} \circ R_{1} }} \left( {a,c} \right) = \min_{b\in B}\left\{ {\max \left[ {f_{{ R_{1} }} \left( {a,b} \right),f_{{ R_{2} }} \left( {b,c} \right)} \right]} \right\};$$

and

$$l_{{ R_{2} \circ R_{1} }} \left( {a,c} \right) =\max_{b\in B} \left\{ {\min \left[ {l_{{ R_{1} }} \left( {a,b} \right),l_{{ R_{2} }} \left( {b,c} \right)} \right]} \right\};$$

$${\text{for}}\;\forall \left( {a,c} \right) \in A \times C.$$

### **Theorem 2**

*The composition of Turiyam relations is associative and invertible.*

### *Proof*

Let $R_{1} :A \to B$, $R_{2} :B \to C$ and $R_{3} :C \to D$ be Turiyam relations on $U.$ First let us show associativity i.e., $(R_{1} \circ R_{2} ) \circ R_{3} = R_{1} \circ (R_{2} \circ R_{3} )$. Let (a, d)$\in A \times D.$ For liberal function we have,

$$\begin{aligned} l_{{R_{1} \circ (R_{2} \circ R_{3} )}} \left( {a,d} \right) & =\max_{c\in C} \left\{ {l_{{R_{2} \circ R_{1} }} \left( {a,c} \right) \wedge l_{{R_{1} }} \left( {c,d} \right)} \right\} \\ & = \max_{c\in C} \left\{ {\max_{b\in B} \left( {l_{{R_{1} }} \left( {a,b} \right) \wedge l_{{R_{2} }} \left( {b,c} \right)} \right) \wedge l_{{R_{1} }} \left( {c,d} \right)} \right\} \\ & =\max_{b\in B} \left\{ {l_{{R_{1} }} \left( {a,b} \right) \wedge \max_{c\in C} \left(( {l_{{R_{2} }} \left( {b,c} \right) \wedge l_{{R_{1} }} \left( {c,d} \right)} \right)} \right\} \\ & =\max_{b\in B} \left\{ {l_{{R_{1} }} \left( {a,b} \right) \wedge l_{{R_{1} \circ R_{2} }} \left( {b,d} \right)} \right\} \\ & = l_{{(R_{1} \circ R_{2} ) \circ R_{3} }} \left( {a,d} \right) \\ \end{aligned}$$

Similarly, we prove truth, indeterminacy, and falsity functions. Hence, the composition of Turiyam relations is associative. Also, easily we can show that $(R_{1} \circ R_{2} )^{ - 1} = R_{2}^{ - 1} \circ R_{1}^{ - 1}$.

## Types of Turiyam relations

We now introduce Turiyam relations types like reflexive, symmetric, transitive and equivalence relation. Also, we derive some properties related to them.

### **Definition 11**

Let $R$ be a Turiyam relation on $U.$

- R is reflexive if $\left( {x,x} \right) \in R,\forall x \in U$
- R is symmetric if $R\left( {x,y} \right) = R \left( {y,x} \right)$
- R is transitive if $R \circ R \subseteq R$ i.e., $\left( {a,b} \right)$ and $\left( {b,c} \right) \in R \Rightarrow \left( {a,c} \right) \in R$
- R is an equivalence relation if it is reflexive, symmetric and transitive at the same time.

### *Example 5*

In Example 2, since $R_{1}$ is reflexive, symmetric and transitive then it is an equivalence relation.

### **Theorem 3**

*The inverse, intersection, union and composition of reflexive Turiyam relations are reflexive.*

### *Proof*

Let $R_{1}$ and $R_{2}$ be two reflexive Turiyam relations on universe set U. Then, $t_{{R_{1} }} \left( {x,{\text{x}}} \right) = i_{{R_{1} }} \left( {x,{\text{x}}} \right) = f_{{R_{1} }} \left( {x,{\text{x}}} \right) = 0, l_{{R_{1} }} \left( {x,{\text{x}}} \right) = 1$ and $t_{{R_{2} }} \left( {x,{\text{x}}} \right) = i_{{R_{2} }} \left( {x,{\text{x}}} \right) = f_{{R_{2} }} \left( {x,{\text{x}}} \right) = 0\;{\text{and}}\; l_{{{\text{R}}_{2} }} \left( {x,{\text{x}}} \right) = 1.$ Clearly, $R_{1}^{ - 1}$ is reflexive relation. To show $R_{1} \cup R_{2}$ is reflexive.

Now $t_{{R_{1} \cup R_{2} }} \left( {x,{\text{x}}} \right) = t_{{R_{1} }} \left( {x,{\text{x}}} \right) \vee t_{{R_{2} }} \left( {x,{\text{x}}} \right) = 0 \vee 0 = 0$,$i_{{R_{1} \cup R_{2} }} \left( {x,{\text{x}}} \right) = i_{{R_{1} }} \left( {x,{\text{x}}} \right) \wedge i_{{R_{2} }} \left( {x,{\text{x}}} \right) = 0 \wedge 0 = 0$, $f_{{R_{1} \cup R_{2} }} \left( {x,{\text{x}}} \right) = f_{{R_{1} }} \left( {x,{\text{x}}} \right) \wedge f_{{R_{2} }} \left( {x,{\text{x}}} \right) = 0 \wedge 0 = 0$ and $l_{{R_{1} \cup R_{2} }} \left( {x,{\text{x}}} \right) = l_{{R_{1} }} \left( {x,{\text{x}}} \right) \vee l_{{R_{2} }} \left( {x,{\text{x}}} \right) = 1 \vee 1 = 1$. Then, $R_{1} \cup R_{2}$ is reflexive. Similarly,$R_{1} \cap R_{2}$ is reflexive.

To show $R_{2} \circ R_{1}$ is reflexive. For $x \in U, l_{{ R_{2} \circ R_{1} }} \left( {x,x} \right) =\max_{y\in U} \left( {l_{{ R_{1} }} \left( {x,y} \right) \wedge l_{{ R_{2} }} \left( {y,x} \right)} \right)$

$$\begin{aligned} & = \left[ \max_{x\neq y\in U } {\left( {l_{{ R_{1} }} \left( {x,y} \right) \wedge l_{{ R_{{2_{1} }} }} \left( {y,x} \right)} \right)} \right] \vee \left[ { l_{{ R_{1} }} \left( {x,x} \right) \wedge l_{{ R_{2} }} \left( {x,x} \right)} \right] \\ & = \left[\max_{x\neq y\in U } ( {l_{{ R_{1} }} \left( {x,y} \right) \wedge l_{{ R_{{2_{1} }} }} \left( {y,x} \right)}) \right] \vee \left[ { {1} \wedge {1} } \right] = {1} \\ \end{aligned}$$

Similar computation gives, $t_{{ R_{2} \circ R_{1} }} \left( {x,x} \right) = i_{{ R_{2} \circ R_{1} }} \left( {x,x} \right) = f_{{ R_{2} \circ R_{1} }} \left( {x,x} \right) = 0.$ Hence, $R_{2} \circ R_{1}$ is reflexive.

### **Theorem 4**

*Let* $R_{1}$ *and* $R_{2}$ *be two symmetric turiyam relations.*

- $R_{1} ^{ - 1},$ $R_{1} \cup R_{2}$ *and* $R_{1} \cap R_{2}$ *are symmetric relations.*

### *Proof*

First let us prove for $R_{1} ^{ - 1} .$ By definition of inverse, we have $R_{1} ^{ - 1} \left( {x,y} \right) = R\left( {y,x} \right) = R\left( {x,y} \right) = R_{1} ^{ - 1} \left( {y,x} \right).$ Then, $R_{1} ^{ - 1}$ is symmetric. Since $R_{1}$ and $R_{2}$ are symmetric, $t_{{R_{1} \cap R_{2} }} \left( {x,{\text{y}}} \right) = t_{{R_{1} }} \left( {x,{\text{y}}} \right) \wedge t_{{R_{2} }} \left( {x,{\text{y}}} \right) = t_{{R_{1} }} \left( {y,{\text{x}}} \right) \wedge t_{{R_{2} }} \left( {y,{\text{x}}} \right)=t_{{R_{1} \cap R_{2} }} \left( {y,{\text{x}}} \right)$,${i_{R_{1} \cap R_{2} }} \left( {x,{\text{y}}} \right) = i_{{R_{1} }} \left( {x,{\text{y}}} \right) \vee i_{{R_{2} }} \left( {x,{\text{y}}} \right) = i_{{R_{1} }} \left( {y,{\text{x}}} \right) \vee i_{{R_{2} }} \left( {y,{\text{x}}} \right)=i_{{R_{1} \cap R_{2} }} \left( {y,{\text{x}}} \right)$, ${f_{R_{1} \cap R_{2} }} \left( {x,{\text{y}}} \right) = f_{{R_{1} }} \left( {x,{\text{y}}} \right) \vee f_{{R_{2} }} \left( {x,{\text{y}}} \right) = f_{{R_{1} }} \left( {y,{\text{x}}} \right) \vee f_{{R_{2} }} \left( {y,{\text{x}}} \right)=f_{{R_{1} \cap R_{2} }} \left( {y,{\text{x}}} \right)$ and $l_{{R_{1} \cap R_{2} }} \left( {x,{\text{y}}} \right) = l_{{R_{1} }} \left( {x,{\text{y}}} \right) \wedge l_{{R_{2} }} \left( {x,{\text{y}}} \right) = l_{{R_{1} }} \left( {y,{\text{x}}} \right) \wedge l_{{R_{2} }} \left( {y,{\text{x}}} \right)=l_{{R_{1} \cap R_{2} }} \left( {y,{\text{x}}} \right)$. Then, $R_{1} \cap R_{2}$ is symmetric. Similarly, we can show $R_{1} \cup R_{2}$ is symmetric.

(b) $R_{1}$ is symmetric if and only if $R_{1} ^{ - 1} = R_{1}$

### *Proof*

It is clear.

(c) $R_{2} \circ R_{1}$ is symmetric if and only if $R_{2} \circ R_{1} = R_{1} \circ R_{2}$

### *Proof*

Let $R_{2} \circ R_{1}$ be symmetric Turiyam relation. Then, $l_{{ R_{2} \circ R_{1} }} \left( {x,y} \right) = l_{{ R_{2} \circ R_{1} }} \left( {y,x} \right)$

$$\begin{aligned} & = \max_{b \in B} \left( {l_{{ R_{1} }} \left( {y,b} \right) \wedge l_{{ R_{2} }} \left( {b,x} \right)} \right) \\ & = \max_{b \in B} \left( {l_{{ R_{2} }} \left( {b,x} \right) \wedge l_{{ R_{1} }} \left( {y,b} \right)} \right) \\ & = \max_{b \in B} \left( {l_{{ R_{2} }} \left( {x,b} \right) \wedge l_{{ R_{1} }} \left( {b,y} \right)} \right) \\ & = l_{{ R_{1} \circ R_{2} }} \left( {x,y} \right) \\ \end{aligned}$$

Similarly, $t_{{ R_{2} \circ R_{1} }} \left( {x,y} \right) = t_{{ R_{1} \circ R_{2} }} \left( {x,y} \right)$, $i_{{ R_{2} \circ R_{1} }} \left( {x,y} \right) = i_{{ R_{1} \circ R_{2} }} \left( {x,y} \right)$ and $f_{{ R_{2} \circ R_{1} }} \left( {x,y} \right) = f_{{ R_{1} \circ R_{2} }} \left( {x,y} \right)$. Thus, $R_{2} \circ R_{1} = R_{1} \circ R_{2} .$ The converse also proved in the same manner. Hence, the proof is valid.

### **Theorem 5**

*Let* $R_{1}$ *and* $R_{2}$ *be two transitiveTuriyam relations. Then,*

- $R_{1} ^{ - 1} , R_{1} \cap R_{2}$ *and* $R_{1}^{2}$ *are also transitive*
- $R_{1} \cup R_{2}$ *is not transitive*

### *Proof*

Let $R_{1}$ and $R_{2}$ be two transitive turiyam relations. Then, (i) it is clear that $R_{1} ^{ - 1}$ is also transitive. (ii) To prove $R_{1} \cap R_{2}$ is transitive, we have

$$\begin{aligned} t_{{(R_{1} \cap R_{2} ) \circ (R_{1} \cap R_{2} )}} \left( {x,y} \right) & = \max_{z \in U} \left\{ {\min \left[ {t_{{R_{1} \cap R_{2} }} \left( {x,z} \right),t_{{R_{1} \cap R_{2} }} \left( {z,y} \right)} \right]} \right\} \\ & = \max_{z \in U}\left\{ {\min \left[ {t_{{R_{1} }} \left( {x,z} \right) \wedge t_{{R_{2} }} \left( {x,z} \right),t_{{R_{1} }} \left( {z,y} \right) \wedge t_{{R_{2} }} \left( {z,y} \right)} \right]} \right\} \\ & = \min \left\{ {\mathop {\max }\limits_{z \in U} \left[ {t_{{R_{1} }} \left( {x,z} \right) \wedge t_{{R_{2} }} \left( {x,z} \right)} \right],\left[ {t_{{R_{1} }} \left( {z,y} \right) \wedge t_{{R_{2} }} \left( {z,y} \right)} \right]} \right\} \\ & = \, \min \left\{ {t_{{R_{1} \circ R_{1} }} \left( {x,y} \right),t_{{R_{2} \circ R_{2} }} \left( {x,y} \right)} \right\} \le \min \left\{ {t_{{R_{1} }} \left( {x,y} \right),t_{{R_{2} }} \left( {x,y} \right)} \right\} = t_{{R_{1} \cap R_{2} }} \left( {x,y} \right). \\ \end{aligned}$$

This implies $${\text{t}}_{{(R_{1} \cap R_{2} ) \circ (R_{1} \cap R_{2} )}} \left( {x,y} \right) \le t_{{R_{1} \cap R_{2} }} \left( {x,y} \right)$$.

Similarly, $i_{{(R_{1} \cap R_{2} ) \circ (R_{1} \cap R_{2} )}} \left( {x,y} \right) \ge i_{{R_{1} \cap R_{2} }} \left( {x,y} \right), f_{{(R_{1} \cap R_{2} ) \circ (R_{1} \cap R_{2} )}} \left( {x,y} \right) \ge f_{{R_{1} \cap R_{2} }} \left( {x,y} \right)$ and ${l_{(R_{1} \cap R_{2} ) \circ (R_{1} \cap R_{2} )}} \left( {x,y} \right) \le l_{{R_{1} \cap R_{2} }} \left( {x,y} \right)$. Thus, $(R_{1} \cap R_{2} ) \circ (R_{1} \cap R_{2} ) \subseteq (R_{1} \cap R_{2} )$. Hence, $R_{1} \cap R_{2}$ is transitive.

(iii) $l_{{{\text{R}}_{1} \circ R_{1} }} \left( {x,y} \right) =\max_{z \in U} \left\{ {{\text{min}}\left( {l_{{{\text{R}}_{1} }} \left( {x,z} \right),l_{{{\text{R}}_{1} }} \left( {z,y} \right)} \right)} \right\} \ge\max_{z \in U} \left\{ {{\text{min}}\left( {l_{{{\text{R}}_{1} \circ R_{1} }} \left( {x,z} \right),l_{{{\text{R}}_{1} \circ R_{1} }} \left( {z,y} \right)} \right)} \right\} = l_{{{\text{R}}_{1}^{2} \circ R_{1}^{2} }} \left( {x,y} \right)$. Similarly, we can prove that $t_{{{\text{R}}_{1}^{2} \circ R_{1}^{2} }} \left( {x,y} \right) \le t_{{{\text{R}}_{1} \circ R_{1} }} \left( {x,y} \right)$, $i_{{{\text{R}}_{1}^{2} \circ R_{1}^{2} }} \left( {x,y} \right) \le i_{{{\text{R}}_{1} \circ R_{1} }} \left( {x,y} \right)$ and $f_{{{\text{R}}_{1}^{2} \circ R_{1}^{2} }} \left( {x,y} \right) \ge f_{{{\text{R}}_{1} \circ R_{1} }} \left( {x,y} \right).$ Hence, $R_{1}^{2}$ is also transitive.

To prove $R_{1} \cup R_{2}$ is not transitive; easily we can show that $t_{{(R_{1} \cup R_{2} ) \circ (R_{1} \cup R_{2} )}} \left( {x,y} \right) \ge$ max $\left\{ {t_{{R_{1} }} \left( {x,y} \right),t_{{R_{2} }} \left( {x,y} \right)} \right\}$.

### **Definition 12**

Let $R$ be Turiyam relations on $U$ and let $x \in U.$ Then the Turiyam equivalence class of x by R (denoted by R[x]) is a Turiyam set in U given by

$$R\left[ x \right] = \left( {t_{R\left[ x \right] } , i_{R\left[ x \right] } ,f_{R\left[ x \right] } ,l_{R\left[ x \right] } } \right)$$

where $t_{{{\text{R}}\left[ {\text{x}} \right]}} , i_{{{\text{R}}\left[ {\text{x}} \right]}} ,f_{{{\text{R}}\left[ {\text{x}} \right]}} ,l_{{{\text{R}}\left[ {\text{x}} \right]}} :U \to \left[ {0,1} \right]$ such that $t_{{{\text{R}}\left[ {\text{x}} \right]}} \left( y \right) = t_{{\text{R}}} \left( {x,y} \right),i_{{{\text{R}}\left[ {\text{x}} \right]}} \left( y \right) = i_{{\text{R }}} \left( {x,y} \right),f_{{{\text{R}}\left[ {\text{x}} \right]}} \left( y \right) = f_{{\text{R }}} \left( {x,y} \right)$ and $l_{{{\text{R}}\left[ {\text{x}} \right]}} \left( y \right) = l_{{\text{R }}} \left( {x,y} \right)$.

### *Example 6*

Consider $R_{2}$ in Example 3.

The set $R_{2} \left[ b \right] = \left\{ {\left\langle {a,\left( {0.1, 0.3, 0.2, 0.4} \right)} \right\rangle ,\left\langle {b,\left( {0.0, 0.2, 0.1, 0.3} \right)} \right\rangle } \right\}$ is the Turiyam equivalence class of b by $R_{2}$.

## Conclusion

This manuscript developed Turiyam relations as the generalization of neutrosophic relations. Union, intersection and composition of Turiyam relations are discussed. Finally, some types of Turiyam relations like reflexive, symmetric, and transitive are defined and some concerning results are derived as desired.

The future work will be, based on the introduced concepts, to apply Turiyam relations in real life situations. Furthermore, Turiyam graphs will be developed and the Turiyam graphs will solve many real life problems where the uncertainty is beyond true, false and indeterminacy.

## Limitations

This study is limited to developing Turiyam Cartesian products and Turiyam relations. The authors study some properties of Turiyam relations. The study focused on the theoretical part of Turiyam relations.

## Data Availability

Not applicable.
